# Reliability and Validity of the Japanese Version of the Inventory of Psychotic‐Like Anomalous Self‐Experiences

**DOI:** 10.1002/mpr.70099

**Published:** 2026-07-19

**Authors:** Dan Nakamura, Masafumi Mizuno, David Cicero, Yu Arai, Hirohisa Suzuki, Shunsuke Hirose, Takahisa Kasai, Shutaro Sugita, Yoshifumi Nakamura, Stephen Wood, Andrea Polari, Takahiro Nemoto, Barnaby Nelson

**Affiliations:** ^1^ Department of Psychiatry Showa Medical University School of Medicine Tokyo Japan; ^2^ Orygen The National Centre of Excellence in Youth Mental Health Melbourne Australia; ^3^ Department of Psychiatry Asaka Hospital Fukushima Japan; ^4^ Department of Psychology University of North Texas Denton Texas USA; ^5^ Department of Neuropsychiatry Toho University Faculty of Medicine Tokyo Japan; ^6^ Centre for Youth Mental Health The University of Melbourne Melbourne Australia; ^7^ School of Psychology University of Birmingham Edgbaston UK; ^8^ Centre for Mental Health and Brain Sciences School of Health Sciences Swinburne University of Technology Melbourne Australia; ^9^ Department of Psychiatry and Implementation Science Toho University Faculty of Medicine Tokyo Japan

**Keywords:** basic self‐disturbance, inventory of psychotic‐like anomalous self‐experiences, schizophrenia

## Abstract

**Objectives:**

Basic self‐disturbance is an abnormality in subjective self‐experience and a core feature of schizophrenia. The Inventory of Psychotic‐Like Anomalous Self‐Experiences (IPASE) is a self‐report measure designed to assess basic self‐disturbance. Despite its clinical utility, a Japanese version has not yet been established. This study aimed to develop a Japanese version of the IPASE and evaluate its reliability and validity.

**Methods:**

The study included 182 participants (83 individuals with schizophrenia and 99 healthy controls). The Japanese version of the IPASE was administered to all participants, along with the PANSS and YMRS, to assess validity.

**Result:**

All IPASE domain scores and the total score were significantly higher in the schizophrenia group than in healthy controls. The IPASE total score showed good internal consistency (Cronbach's *α* > 0.80). In the schizophrenia group, the PANSS positive subscale, general psychopathology subscale, and total scores were all significantly positively correlated with IPASE scores. These positive correlations with the positive subscale support convergent validity. ROC analysis indicated that the IPASE total score had good discriminative ability for schizophrenia diagnosis (AUC = 0.851).

**Conclusion:**

The Japanese version of the IPASE demonstrated high reliability and validity.

## 1 Introduction

Basic self‐disturbance (also termed disturbances of the minimal self, ipseity disturbances (Sass and Parnas 2003), or Anomalous Self‐Experiences (ASEs) (Parnas and Handest 2003) is an abnormality in subjective self‐experience and a core feature of schizophrenia (Nelson et al. 2015). The ‘basic self’ refers to the pre‐reflective and immediate consciousness of experience, thought, and action, encompassing a sense of ownership and agency over one's experiences and actions (Gallagher 2011; Zahavi and Gallagher 2020). When the basic self destabilizes, individuals may experience a range of anomalous subjective experiences, including a diminished sense of ownership over thoughts or bodily experiences, disturbances in self‐identity, and alterations in first‐person perspective, sometimes described as observing oneself from a distance. Such experiences often involve significant distress. Basic self‐disturbance has also been reported in individuals at clinical high risk for psychosis (CHR‐P) (Nelson et al. 2012) and may increase over time, potentially contributing to the development of positive and negative symptoms (Parnas and Handest 2003). These experiences often lead to confusion, disorientation, and difficulties in social functioning and understanding (“common sense”), frequently accompanied by significant distress (Nelson et al. 2015).

In 2005, the Examination of Anomalous Self‐Experiences (EASE), a semi‐structured interview designed to assess basic self‐disturbance, was developed (Parnas et al. 2005a). The availability of a standardized instrument for assessing basic self‐disturbance led to a growing body of research on this construct. The EASE has demonstrated high internal consistency, inter‐rater reliability, and temporal stability (Parnas et al. 2005b). Research using the EASE indicates that basic self‐disturbance occurs frequently in schizophrenia (Parnas et al. 2005b; Nordgaard et al. 2021; Haug et al. 2012) and is more prominent than in other psychotic disorders, such as bipolar disorder with psychosis (Henriksen et al. 2021a, 2021b). It also predicts future onset of schizophrenia in CHR‐P groups (Nelson et al. 2012). Furthermore, it is significantly associated with positive and negative symptoms in both schizophrenia and CHR‐P (Nelson et al. 2012; Parnas et al. 2005b; Søndergaard et al. 2014; Schultze‐Lutter et al. 2016) and linked to impairments in social cognition (Søndergaard et al. 2014; Nelson et al. 2009). The EASE has been translated into 10 languages and provides a comprehensive assessment of basic self‐disturbance. However, its administration requires approximately 90 minutes, and interviewers must complete rigorous certification training, limiting its use to research settings and posing challenges for clinical application. In response, the Inventory of Psychotic‐Like Anomalous Self‐Experiences (IPASE) was developed as a simpler, shorter assessment suitable for clinical use (Cicero et al. 2017). Each IPASE item was based on phenomenological descriptions of past basic self‐disturbance with reference to the EASE. Because these items are not direct translations of EASE items, the IPASE should not be considered a shortened version of the EASE self‐report scale. As a self‐report questionnaire, responses may be influenced by the respondent's introspective ability, self‐understanding, and subjective interpretation (Henriksen et al. 2021a, 2021b; Cicero et al. 2017). The IPASE is not intended to replace clinical interviews using the EASE but is primarily considered a screening measure for basic self‐disturbance (Nelson et al. 2019). IPASE scores were significantly higher in the schizophrenia group compared to healthy controls and demonstrated high internal reliability, with Cronbach's alpha coefficients ranging from 0.96 to 0.98 (Cicero et al. 2017). Additionally, the IPASE correlated strongly with the standardized tool for evaluating CHR‐P, the Comprehensive Assessment of At‐Risk Mental States (CAARMS), particularly with its positive symptom score (Nelson et al. 2019), supporting its convergent validity. Although not as precise as the EASE, the IPASE is useful in predicting future psychotic episodes in CHR‐P populations (Nelson et al. 2012). Recent studies using the IPASE have reported on the neurocognitive basis of basic self‐disturbance. Roig‐Herrero et al. found that IPASE scores positively correlated with functional connectivity between specific components of the Default Mode Network—a series of brain regions associated with internal thoughts and self‐referential processing (Roig‐Herrero et al. 2022).

The concept of basic self‐disturbance was systematized in Europe, where the longstanding tradition of psychopathology has emphasized qualitative changes in self‐experience, leading to advanced research on phenomenological approaches to self‐experience disturbances (Mishara et al. 2014; de Vries et al. 2013). In contrast, Japanese schizophrenia research, initially influenced by German psychiatry, became predominantly shaped by American psychiatry, focusing on external manifestations of clinical symptoms such as hallucinations and delusions (Utena and Niwa 1992). This trend intensified after the introduction of operational diagnostic criteria in DSM‐III (Sato and Berrios 2001), and interest in self‐disturbances remained limited for an extended period. Due to these cultural differences, Japanese versions of the EASE and IPASE have not yet been translated. Recently, while the DSM's operational diagnostic criteria have contributed to clinical standardization, criticism has emerged that they fail to adequately capture the subtle transformations in subjective experience and abnormalities in self‐perception observed in schizophrenia (Pickersgill 2014; Stein et al. 2022). Consequently, the complexity of psychopathology and the evaluation of subtle changes in early onset may be insufficiently addressed. Therefore, assessing basic self‐disturbance in schizophrenia and CHR‐P is critically important for understanding pathologies not captured by conventional diagnostic systems and enabling early clinical intervention. The IPASE, due to its accessibility and simplicity of administration, appears to be an ideal candidate for Japanese translation and utilization.

The study aimed to translate the original IPASE into Japanese and evaluate its reliability and validity in a sample of Japanese patients with schizophrenia compared to healthy controls.

## 2 Materials and Methods

### 2.1 Procedure

Before evaluating the reliability and validity of the Japanese version of IPASE, permission to develop it was obtained from Professor David Cicero, the original author of the IPASE. The translation followed internationally recognized procedures for linguistic validation (Wild et al. 2005). The original version was first forward‐translated into Japanese. An experienced translator then performed a back‐translation, which Professor Cicero reviewed to confirm conceptual equivalence with the original scale. Items identified as conceptually different from the original were revised to ensure consistency. Additionally, Japanese‐speaking clinicians conducted reviews with healthy individuals, and two psychiatrists with extensive clinical experience evaluated the Japanese version, confirming its consistency with the original and appropriateness of the items from a clinical perspective. Cognitive debriefing confirmed that the translated items were understandable to the general public, completing the Japanese version of IPASE.

### 2.2 Participants

In total, 182 individuals participated in this study, comprising 83 patients with schizophrenia (mean age: 24.67 years, standard deviation [SD]: 3.80; 43 males and 40 females), diagnosed according to DSM‐5 criteria and aged 15–30 years, and 99 healthy controls (mean age: 24.65 years, SD: 3.46; 51 males and 48 females). Participants with schizophrenia were recruited from inpatients or outpatients at Showa Medical University Karasuyama Hospital and Toho University Medical Center Omori Hospital in Tokyo, Japan, between January and September 2025. Exclusion criteria for the schizophrenia group included: (a) age younger than 15 or older than 30 years, and (b) severe hallucinatory–delusional states requiring physical restraint or impulsive behaviors necessitating strict safety measures. Healthy controls were recruited during the same period through hospital staff and acquaintances. Their exclusion criteria were: (a) age younger than 15 or older than 30 years, (b) presence of a mental disorder according to DSM‐5 criteria, and (c) history of mental disorders or psychiatric outpatient treatment.

Diagnoses were confirmed by two clinicians with extensive experience in schizophrenia, based on various information sources, including interactions during diagnostic assessments and the participants' past psychiatric and medical histories. Final diagnoses were determined by unanimous agreement of two psychiatrists based on DSM‐5 criteria. Healthy participants were interviewed by psychiatrists, confirming no current mental disorders, no history of past mental illness, and no history of psychiatric outpatient treatment. All participants were native Japanese speakers. As compensation for their participation, participants received a 3000‐yen QUO card as compensation for their participation in this study.

### 2.3 Rating Scale

Following the diagnostic procedures described above, the Positive and Negative Syndrome Scale (PANSS) and the Young Mania Rating Scale (YMRS) were administered to all participants, in addition to the IPASE, to examine the reliability and validity of the IPASE. The PANSS, which assesses the severity of schizophrenia symptoms, was used to evaluate convergent validity. The YMRS, a clinical scale for assessing the severity of manic symptoms, served as an indicator of discriminant validity, as it measures constructs distinct from the schizophrenia symptoms addressed in this study. Assessments were typically conducted at Showa Medical University Karasuyama Hospital or Toho University Medical Center Omori Hospital. All assessments, including IPASE, PANSS and YMRS were administered on the same day. However, for healthy participants unable to visit the hospital, the PANSS and YMRS were administered remotely via an online video conferencing platform, while the IPASE was mailed for self‐administration.

1. Inventory of Psychotic‐Like Anomalous Self‐Experiences (IPASE)
  The IPASE is a self‐report questionnaire consisting of 57 items where participants indicate their level of agreement with statements on a scale of 1 (Strongly Disagree) to 5 (Strongly Agree) (Cicero et al. 2017). Each item is developed from phenomenological descriptions of basic self‐disturbance and is classified into five subscales: Cognition, Self‐Awareness and Presence, Consciousness, Somatization, and Demarcation/Transitivism. The Cognition subscale includes items related to disturbances in thought processes, such as thought interference. The Self‐Awareness and Presence subscale comprises items related to the loss of basic self, identity, and attunement with the world. The Consciousness domain encompasses disturbances in temporal experience and intentionality, as well as difficulties distinguishing imagination from reality. The Somatization domain includes experiences of bodily deformation, disturbance of bodily control, and the sensation of not existing within one's own body. The Demarcation/Transitivism subscale involves diminished self‐world boundaries and experiences of self‐annihilation. By calculating scores for each subscale and the total score, the severity of basic self‐disturbance can be assessed.
2. Positive and Negative Syndrome Scale (PANSS)
  The PANSS is an assessment scale designed to comprehensively evaluate schizophrenia symptoms (Kay et al. 1987). It consists of 30 items: 7 positive symptoms, 7 negative symptoms, and 16 general psychopathology items. Each item is rated on a 7‐point scale (1 = absent, 7 = extreme), with higher scores indicating more severe symptoms. This study utilized the Japanese version of the PANSS, which has established reliability and validity (Yamada 1993; Igarashi et al. 1998). The PANSS was administered by trained raters, who evaluated participants based on semi‐structured interviews and observational information.
3. Young Mania Rating Scale (YMRS)
  The YMRS is an interview‐based rating scale developed for the clinical assessment of manic episodes (Young et al. 1978). It consists of 11 items: elevated mood, increased motor activity/energy, sexual interest, sleep, irritability, speech (rate and amount), language–thought disorder, thought content, disruptive–aggressive behavior, appearance, and insight. Four items—irritability, speech, thought content, and disruptive–aggressive behavior—are rated on a scale from 0 to 8 in 2‐point increments, with weighted scoring to reflect severity. The remaining seven items are rated on a scale from 0 to 4, with 1‐point increments, yielding a total score of 0–60. In this study, the Japanese version of the YMRS, which has demonstrated reliability (Inada et al. 2012), was used. The YMRS was administered by trained raters, who evaluated participants based on behavioral observations during the interview and subjective reports.

### 2.4 Statistical Analysis

#### 2.4.1 Sample Size

Previous studies examining the minimum sample size required for the translation of self‐report symptom assessment scales indicate that 160–200 participants are necessary to ensure validity (Colin and Derek 2018; Colin and Caroline 2017; Marlene et al. 2007). Given these findings and the relatively large number of items in the IPASE (57), a minimum sample size of 180 participants was deemed necessary for the present study.

#### 2.4.2 Demographic and Clinical Characteristics of Schizophrenia and Healthy Control Groups

The schizophrenia and healthy control groups were compared on demographic variables (age, sex, and years of education), PANSS subscale and total scores, YMRS item and total scores, and IPASE item and total scores. Clinical data collected from the schizophrenia group included age at onset, duration of illness, duration of untreated illness, number of hospitalizations, and antipsychotic medication dosage. Independent *t*‐tests and Chi‐square tests were used for continuous and categorical variables, respectively.

#### 2.4.3 Internal Consistency

The internal consistency of the IPASE was assessed using Cronbach's alpha (Cortina 1993; Taber 2018), which measures average inter‐item correlation within the scale. Cronbach's alpha coefficients were calculated for each of the five IPASE subscales and the total scale, separately for the schizophrenia and healthy control groups and for the overall sample. A Cronbach's alpha value of 0.70 or higher is generally considered acceptable; thus, values ≥ 0.70 indicate acceptable internal consistency for the total IPASE and each subscale.

#### 2.4.4 Construct Validity

To examine the construct validity of the IPASE, Pearson's correlation coefficients were calculated among measures administered to the schizophrenia group. For convergent validity, correlations were computed between the IPASE subscale and total scores and the PANSS subscale and total scores, interpreted as evidence supporting the construct validity of the IPASE. For discriminant validity, correlations were calculated between the IPASE subscale and total scores and the YMRS total score. The significance level was set at *p* < 0.01. Correlation coefficients (*r*) were interpreted as follows: < 0.20: little, 0.20–0.40: weak, 0.40–0.70: moderate, 0.70–1.0: strong.

#### 2.4.5 Logistic Regression Analysis

To examine the predictive validity of the IPASE in distinguishing participants with a clinical diagnosis of schizophrenia, a logistic regression analysis was performed with the IPASE total score as the predictor. Additionally, to determine the relative strength of the subscales in classifying schizophrenia and healthy controls, all five subscales were entered stepwise into a logistic regression analysis (Hosmer et al. 2013). The odds ratio (OR) indicates the extent to which increases in the IPASE total score are associated with the likelihood of a clinical diagnosis of schizophrenia.

#### 2.4.6 ROC Analysis

We examined the receiver operating characteristic (ROC) curve using the IPASE total score to analyze the IPASE's accuracy in classifying participants into DSM‐5 diagnostic categories. An area under the curve (AUC) of 1 indicates perfect sensitivity and specificity, while 0.5 indicates a test that is entirely ineffective in discriminating diagnostic status. AUCs are interpreted as excellent (0.90–1.00), good (0.80–0.90), fair (0.70–0.80), poor (0.60–0.70), and bad (0.50–0.60) (Metz 1978).

All statistical analyses were conducted using SPSS version 29.0 software (IBM Corp., Armonk, NY, USA). Statistical significance was set at 0.05, except for correlations, which were set at 0.01 to mitigate the risk of type I error.

## 3 Results

### 3.1 Demographics, Positive and Negative Syndrome Scale Scores, and Young Mania Rating Scale for Schizophrenia and Healthy Control Groups

The demographic and clinical characteristics of the schizophrenia and healthy control groups are presented in Table 1. No significant differences were observed between groups in gender or mean age; however, the healthy control group had a significantly longer educational duration. In the schizophrenia group, Autism Spectrum Disorder (ASD) and Attention‐Deficit/Hyperactivity Disorder (ADHD) were the most common comorbid conditions. The PANSS total score and all subscale scores were significantly higher in the schizophrenia group. For the YMRS, the total score and several item scores, including “Sexual interest” and “Irritability,” were also significantly higher in the schizophrenia group.

**TABLE 1:** Demographics, positive and negative syndrome scale scores, and young mania rating scale scores for schizophrenia and healthy control group.

	Schizophrenia (*N* = 83)		Healthy control (*N* = 99)		*p*‐value
	Mean (SD)	Range	Mean (SD)	Range	
Sex (M, F)	(43, 40)		(51, 48)		0.969
Age, years	24.67 (3.80)	16–30	24.65 (3.46)	15–30	0.958
Years of education	13.30 (2.29)	9–18	15.98 (2.19)	8–21	< 0.001**
Age at onset	18.39 (3.93)	10–28	(−)	(−)	(−)
Duration of illness (years)	6.68 (4.12)	0.42–20	(−)	(−)	(−)
Duration of untreated illness (years)	1.20 (2.04)	0–9	(−)	(−)	(−)
Number of hospitalizations	1.66 (1.43)	0–6	(−)	(−)	(−)
Dosage of antipsychotics:CP equivalent (mg)	633.33 (409.03)	37.88–1709.09	(−)	(−)	(−)
Comorbidities: *N* (%)^a^					
Total	23 (27.71)	(−)	(−)	(−)	(−)
ASD	10 (12.05)	(−)	(−)	(−)	(−)
ADHD	10 (12.05)	(−)	(−)	(−)	(−)
SUD	3 (3.61)	(−)	(−)	(−)	(−)
OCD	2 (2.41)	(−)	(−)	(−)	(−)
PANSS: Positive scale	16.18 (5.12)	7ー28	7.77 (1.11)	7ー14	< 0.001**
PANSS: Negative scale	21.02 (6.81)	7ー39	8.28 (1.82)	7ー14	< 0.001**
PANSS: General psychopathology scale	36.92 (10.52)	16ー67	18.43 (2.40)	16ー30	< 0.001**
PANSS: Total	74.12 (20.18)	30ー134	34.48 (4.15)	30ー51	< 0.001**
YMRS:1 elevated mood	0.34 (0.72)	0–3	0.13 (0.37)	0–2	0.020*
YMRS:2 increased motor activity‐energy	0.23 (0.45)	0–2	0.13 (0.34)	0–1	0.106
YMRS:3 sexual interest	0.13 (0.38)	0–2	0.03 (0.17)	0–1	0.024*
YMRS:4 sleep	0.20 (0.56)	0–2	0.12 (0.44)	0–2	0.268
YMRS:5 irritability	0.47 (0.90)	0–4	0.18 (0.58)	0–2	0.013*
YMRS:6 speech (rate and amount)	0.28 (0.87)	0–6	0.10 (0.44)	0–2	0.098
YMRS:7 language‐thought disorder	0.30 (0.60)	0–2	0.07 (0.26)	0–1	0.001*
YMRS:8 content	2.58 (3.33)	0–8	0.36 (0.78)	0–2	< 0.001**
YMRS:9 disrutive‐aggressive behavior	0.22 (0.63)	0–2	0.12 (0.48)	0–2	0.256
YMRS:10 appearance	0.19 (0.45)	0–2	0.01 (0,10)	0–1	< 0.001**
YMRS:11 insight	0.36 (0.69)	0–2	0	0	< 0.001**
YMRS: Total	5.30 (5.18)	0–23	1.27 (2.15)	0–13	< 0.001**

Abbreviations: ADHD, attention deficit hyperactivity disorder; ASD, autism spectrum disorder; F, female; M, male; OCD, obsessive‐compulsive disorder; PANSS, Positive and Negative Syndrome Scale; SD, standard deviation; SUD, substance use disorder; YMRS, Young Mania Rating Scale.

^a^ Prevalence rates for comorbid conditions are presented for each condition separately and may include overlapping cases (e.g., individuals with both ASD and ADHD).

^*^ *p* < 0.05.

^**^ *p* < 0.01 by independent *t*‐test or Chi‐square test.

### 3.2 Comparison of Groups on Domain Scores of the IPASE

**FIGURE 1:**
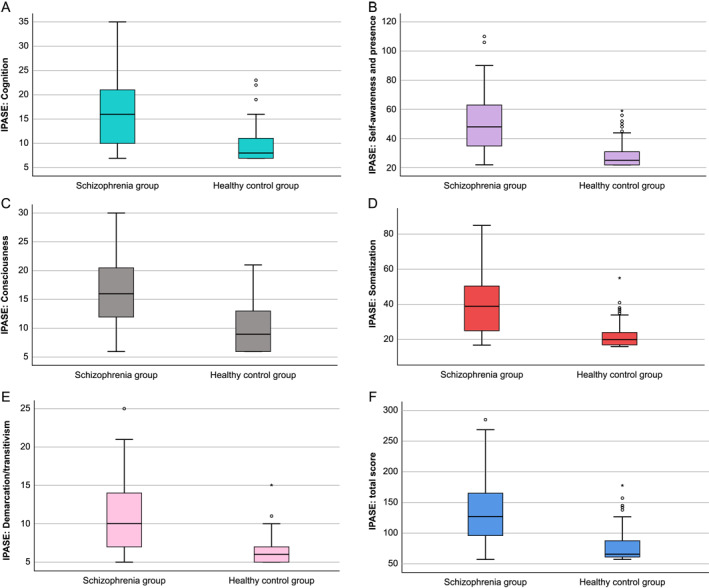
Boxplots of IPASE subscale and total scores in schizophrenia and healthy control groups. Boxplots depict the distribution of IPASE subscales and total scores for individuals with schizophrenia and healthy controls. (A) Cognition scores. (B)Self‐Awareness and Presence scores. (C)Consciousness scores. (D)Somatization scores. (E)Demarcation/Transitivism. (F)Total scores. Boxes represent the interquartile range (IQR), horizontal lines indicate median values, whiskers denote 1.5 × IQR, and outliers are shown as individual points. Significant differences between groups are indicated in the results section (see Table 2 for detailed *t*‐test results).

The scores for each IPASE subscale and the total IPASE score in the schizophrenia and healthy control groups are shown in Table 2. All subscale scores and the total IPASE score were significantly higher in the schizophrenia group than in the healthy control group. Box‐and‐whisker plots (Figure 1) illustrate the distributions of IPASE subscale scores and total scores for both groups. The schizophrenia group exhibited higher median scores and a wider distribution across all domains than the healthy control group, which showed lower scores and a narrower distribution. However, multiple outliers were present in several domains.

**TABLE 2:** Mean domain scores of the inventory of psychotic‐like anomalous self‐experiences in the schizophrenia and healthy control groups.

	Schizophrenia (*N* = 83)		Healthy control (*N* = 99)			
IPASE	Mean (SD)	Range	Mean (SD)	Range	*t*	*p*
Cognition	16.73 (7.22)	7ー35	9.83 (3.42)	7ー23	7.994	< 0.001**
Self‐awareness and presence	50.13 (19.62)	22ー110	28.19 (8.49)	22ー59	9.473	< 0.001**
Consciousness	16.22 (5.80)	6ー30	10.02 (4.11)	6ー21	8.17	< 0.001**
Somatization	39.19 (15.66)	17ー85	22.62 (7.20)	17ー55	8.888	< 0.001**
Demarcation/Transitivism	10.96 (4.38)	5ー25	6.45 (1.92)	5ー15	8.701	< 0.001**
Total	133.24 (49.27)	57ー285	77.12 (22.61)	57ー157	9.567	< 0.001**

Abbreviation: IPASE: Inventory of Psychotic‐Like Anomalous Self‐Experience.

^**^ *p* < 0.01 by independent *t*‐test.

### 3.3 Internal Consistency of the IPASE Total and Each Sub‐Scale in Schizophrenia Group, Healthy Control Group, and Overall Participants

Table 3 presents Cronbach's alpha values for the IPASE total score and each subscale across the schizophrenia group, the healthy control group, and the total sample. For the IPASE total score, Cronbach's alpha exceeded 0.80 in all groups, indicating good internal consistency. For subscales, Cronbach's alpha exceeded 0.80 except for Demarcation/Transitivism in the total sample. Notably, the Self‐Awareness and Presence and Somatization subscales demonstrated Cronbach's alpha values exceeding 0.90, indicating excellent internal consistency. The Demarcation/Transitivism subscale showed low internal consistency in the healthy control group; however, acceptable internal consistency in the total sample.

**TABLE 3:** Cronbach's Alpha for the IPASE total and subscale in the schizophrenia, healthy control group, and overall participants.

	Cronbach's alpha		
IPASE	Schizophrenia	Healthy control	Total
Total	0.871	0.869	0.884
Cognition	0.872	0.749	0.886
Self‐awareness and presence	0.951	0.885	0.964
Consciousness	0.828	0.764	0.858
Somatization	0.925	0.87	0.942
Demarcation/Transitivism	0.759	0.603	0.812

*Note:* Cronbach's alpha coefficients demonstrated the internal consistency of the IPASE total and each scale. Reliability indices were reported for the patients with schizophrenia, healthy controls, and all participants.

### 3.4 Relationship of IPASE Scores With PANSS and YMRS Scores in the Schizophrenia Group

Table 4 presents correlations between IPASE scores (total and subscales) and PANSS and YMRS total scores in the schizophrenia group. The PANSS positive symptom scale score, general psychopathology scale score, and PANSS total score showed significant moderate positive correlations with all IPASE subscale scores and the total score. In contrast, the PANSS negative symptom scale score showed weak positive correlations with the IPASE Self‐Awareness and Presence and Demarcation/Transitivism subscale scores, with no significant correlations observed with other IPASE subscale scores or the total score. Significant correlations were also noted between the YMRS total score and IPASE scores (total and subscales).

**TABLE 4:** Relationship between IPASE, PANSS, and YMRS scores in the schizophrenia group.

	IPASE: Cognition	IPASE: Self‐awareness and presence	IPASE: Consciousness	IPASE: Somatization	IPASE: Demarcation/Transitivism	IPASE: Total
PANSS: Positive scale	0.586^a^	0.592^a^	0.506^a^	0.606^a^	0.473^a^	0.616^a^
PANSS: Negative scale	0.278	0.288^a^	0.238	0.211	0.318^a^	0.279
PANSS: General psychopathology scale	0.566^a^	0.568^a^	0.489^a^	0.508^a^	0.532^a^	0.575^a^
PANSS: Total	0.578^a^	0.585^a^	0.500^a^	0.527^a^	0.543^a^	0.592^a^
YMRS: Total	0.455^a^	0.474^a^	0.342^a^	0.453^a^	0.405^a^	0.476^a^

Abbreviations: IPASE: Inventory of Psychotic‐Like Anomalous Self‐Experiences; PANSS: Positive and Negative Syndrome Scale; YMRS, Young mania rating scale.

^a^ Correlation is significant at the 0.01 level (2‐tailed).

**FIGURE 2:**
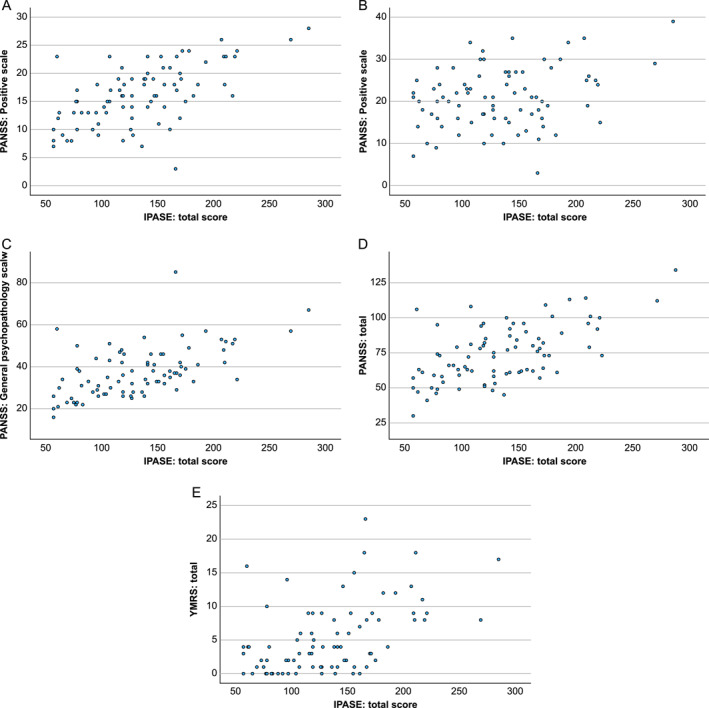
Scatter plot of the correlation between IPASE total score and PANSS subscale, total, and YMRS total scores. The scatter plots show the correlation between the IPASE total score and (A) PANSS positive scale, (B) PANSS negative Subscale, (C) PANSS general psychopathological score, (D) PANSS total score, and (E) YMRS total score for the schizophrenia group. Each plot shows the relationship between the total IPASE score and the respective measure, indicating how these variables are associated within the schizophrenia group. Owing to overlapping data points at identical coordinates, the scatter plot displays fewer points than the actual sample size (*N* = 83).

The relationships between IPASE scores (total and subscale) and the PANSS positive symptom scale, general psychopathology scale, PANSS total score, and YMRS total score are illustrated in the scatter plots in Figure 2. In all plots, data points exhibit an upward trend, indicating that higher IPASE scores are associated with higher PANSS and YMRS scores. This pattern aligns with the correlation analyses, and the strength of the correlations is reflected in the scatter plots.

**FIGURE 3:**
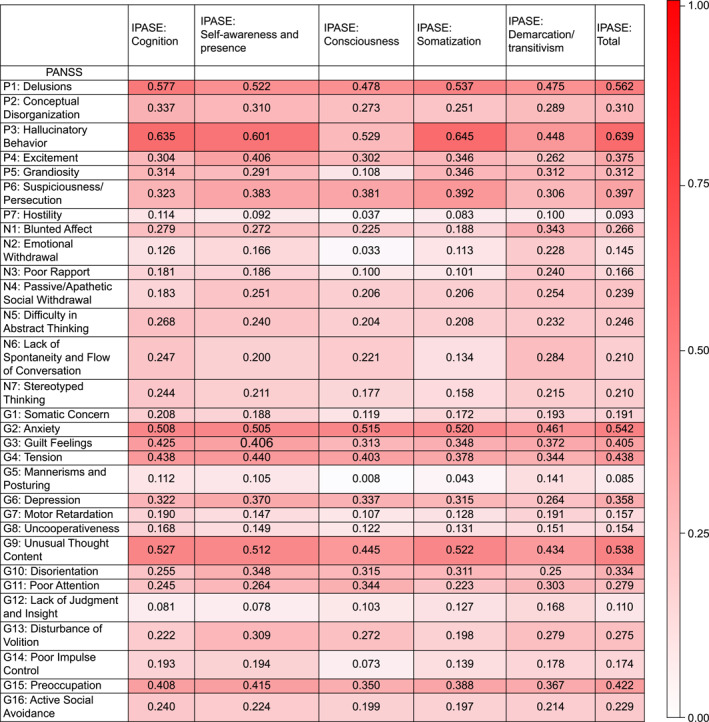
Heatmap of correlations between IPASE total and subscale scores, and individual PANSS item scores in the schizophrenia group. Color intensity represents the strength of the correlation coefficients (r values), with darker red indicating stronger positive correlations. Rows correspond to individual PANSS items (P, N, and G subscales), and columns represent IPASE total and subscale scores.

Correlations between IPASE total and subscale scores and each PANSS item are displayed in the heatmap in Figure 3. Results indicate that IPASE total and subscale scores showed statistically significant moderate positive correlations with PANSS items P1 (Delusions), P3 (Hallucinatory Behavior), G2 (Anxiety), G3 (Guilt Feelings), G4 (Tension), G9 (Unusual Thought Content), and G15 (Preoccupation). Notably, the IPASE total score showed the strongest correlations with P1 and P3. In contrast, none of the PANSS negative scale items (N1–N7) showed significant correlations with the IPASE total score.

Finally, correlations between IPASE total and subscale scores and individual YMRS items are presented in Table 5. The IPASE total score showed statistically significant weak positive correlations with items 3 (Sexual interest), 7 (Language–Thought Disorder), and 8 (Content).

**TABLE 5:** Correlations between IPASE and individual YMRS item scores in the schizophrenia group.

	IPASE: Cognition	IPASE: Self‐awareness and presence	IPASE: Consciousness	IPASE: Somatization	IPASE: Demarcation/Transitivism	IPASE: Total
YMRS						
1. Elevated mood	0.294^a^	0.265	0.146	0.264	0.259	0.273
2. Increased motor Activity‐energy	0.199	0.197	0.032	0.215	0.091	0.188
3. Sexual interest	0.346^a^	0.339^a^	0.284^a^	0.350^a^	0.240	0.352^a^
4. Sleep	0.077	0.129	0.133	0.220	−0.072	0.142
5. Irritability	0.237	0.245	0.155	0.187	0.273	0.234
6. Speech (rate and Amount)	0.190	0.230	0.241	0.181	0.108	0.215
7. Language‐thought Disorder	0.368^a^	0.390^a^	0.283^a^	0.337^a^	0.334^a^	0.379^a^
8. Content	0.339^a^	0.369^a^	0.242	0.351^a^	0.344^a^	0.367^a^
9.Disrutive‐aggressive Behavior	0.029	−0.010	0.000	0.003	−0.042	−0.003
10. Appearance	0.072	0.004	0.072	0.024	0.077	0.035
11. Insight	0.144	0.137	0.160	0.138	0.178	0.154

Abbreviations: IPASE: Inventory of Psychotic‐Like Anomalous Self‐Experiences; PANSS: Positive and Negative Syndrome Scale; YMRS, Young mania rating scale.

^a^ Correlation is significant at the 0.01 level (2‐tailed).

### 3.5 Logistic Regression Analysis

Logistic regression analysis demonstrated that the IPASE total score significantly predicts distinctions between individuals with schizophrenia and healthy controls (OR = 1.045, 95% CI = 1.032–1.059, *p* < 0.001). This indicates that for every one‐unit increase in IPASE scores (ranging from 57 to 285), the likelihood of being in the schizophrenia group increases by 4.5%. In the final model of the stepwise analysis, only the Self‐Awareness and Presence *and* Demarcation/Transitivism subscales were retained (Self‐Awareness and Presence: OR = 1.079, 95% CI = 1.037–1.124, *p* < 0.001; Demarcation/Transitivism: OR = 1.262, 95% CI = 1.062–1.501, *p* = 0.008). These findings suggest that the predictive ability of the IPASE score in distinguishing between individuals with schizophrenia and healthy controls primarily relies on these two subscales.

### 3.6 ROC Analysis

**FIGURE 4:**
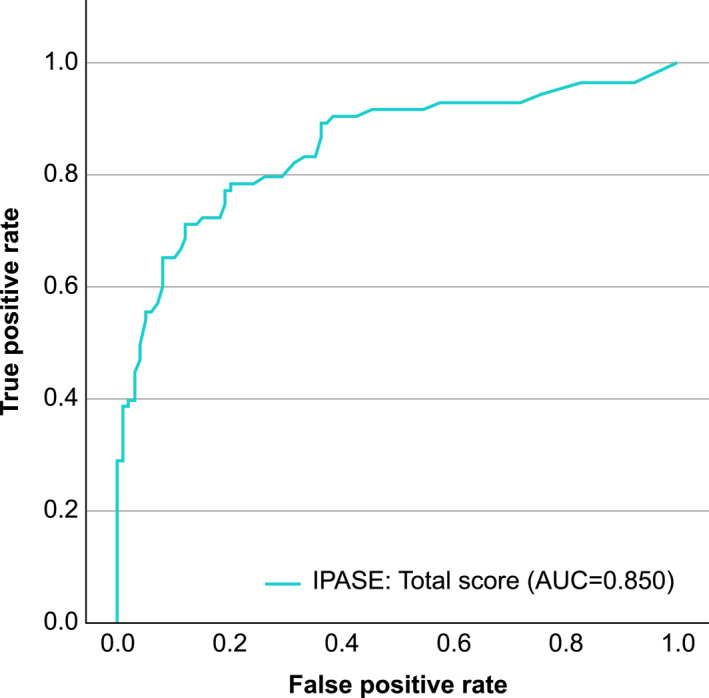
Receiver operating characteristic curves predicting the diagnosis of schizophrenia based on DSM‐5 on the IPASE total score. AUC, Area under the receiver operating characteristic curve; DSM‐5, Diagnostic and Statistical Manual of Mental Illnesses, Fifth Edition; IPASE, Inventory of Psychotic‐Like Anomalous Self‐Experiences.

The ROC for predicting schizophrenia diagnosis based on the IPASE total score is shown in Figure 4. The IPASE total score demonstrated strong discriminative ability in distinguishing individuals with schizophrenia from healthy controls (AUC = 0.851).

## 4 Discussion

In this study, we developed the Japanese version of the IPASE and evaluated its reliability and validity. Internal consistency was assessed to determine reliability. Cronbach's alpha values exceeded 0.80 in the schizophrenia group, the healthy control group, and the total sample, indicating good internal consistency. Construct validity was evaluated by examining correlations with the PANSS and YMRS in the schizophrenia group. Significant moderate positive correlations were observed between the PANSS positive symptom scale, the general psychopathology scale, and the total score, and all IPASE subscales and total scores. Positive correlations with the PANSS positive symptom scale provide convergent evidence supporting construct validity. Additionally, the PANSS general psychopathology scale assesses a wide range of symptoms beyond positive and negative symptoms, including anxiety, tension, and depression. These findings suggest that the Japanese version of the IPASE is related to the overall severity of schizophrenia symptoms. Furthermore, some items in the general psychopathology scale closely resemble those in the positive symptom scale; for instance, *Unusual Thought Content* closely resembles delusions. Therefore, the general psychopathology scale may show a positive correlation with IPASE scores, similar to that of the positive symptom scale. In contrast, no significant correlations were found between the PANSS negative symptom scale score and the IPASE total score or most subscale scores, except for the Self‐Awareness and Self‐Presence and Demarcation/Transitivism subscales. Parnas argued that basic self‐disturbance reflects a qualitative alteration in first‐person self‐experience, whereas negative symptoms represent external behavioral impairments, such as reduced facial expression and social withdrawal. Thus, the two are qualitatively distinct constructs (Parnas and Henriksen 2014). The present findings support this distinction.

Correlations between IPASE scores and each PANSS item revealed significant positive associations with P1 (Delusions), P3 (Hallucinatory behavior), G2 (Anxiety), G3 (Guilt Feelings), G4 (Tension), G9 (Unusual Thought Content), and G15 (Preoccupation). Basic self‐disturbance is suggested to lead to instability in the sense of self and self‐boundaries, contributing to hallucinations and delusions through distortions in reality interpretation and relationships with others (Parnas and Handest 2003; Nordgaard et al. 2014). These findings support this view. In contrast, general symptoms such as anxiety, guilt, and tension have traditionally been considered distinct from basic self‐disturbance (Parnas and Handest 2003; Parnas et al., 2005; Parnas and Henriksen 2014; Henriksen et al. 2021a, 2021b). This study suggests that basic self‐disturbance may be accompanied by emotional distress; however, these constructs are not conceptually identical. Further examination of discriminant validity is needed to determine if IPASE scores are also elevated in conditions like major depressive disorder and anxiety disorders, where these general symptoms are common.

The YMRS total score, used to assess discriminant validity, showed significant, weak‐to‐moderate positive correlations with all IPASE subscales and total scores. Concerns about discriminant validity arise when Pearson's correlation coefficients exceed 0.70–0.80 between scales assessing different constructs (Cheung et al. 2024). The correlations in this study were moderate, suggesting that the two scales assess distinct constructs and that discriminant validity is largely preserved. All participants in the correlational analyses had a diagnosis of schizophrenia, so these findings should be interpreted within that context. These correlations may not be the same in individuals with more severe bipolar symptoms, but this possibility warrants further investigation of discriminant validity by comparing IPASE scores between patients with schizophrenia and those with bipolar disorder. Although the mean YMRS total score in the schizophrenia group (5.30) was significantly higher than in the healthy control group, previous studies have defined YMRS scores below 12 in adolescent bipolar disorder as indicating “minimal or no mania” (Miklowitz et al. 2008). Thus, while the observed difference was statistically significant, the score did not indicate a clinically meaningful manic state, and symptoms remained within the subclinical range.

In addition, the mean IPASE total score in this study was 133.24, comparable to those reported in previous studies of individuals with schizophrenia (130.22 (Hernández‐García et al. 2021, 135.16 Ballerini et al. 2023)). IPASE scores were significantly higher in the schizophrenia group than in healthy controls across all subscale and total scores. The ROC‐AUC for predicting schizophrenia diagnosis using the IPASE total score was 0.851, indicating good discriminative ability. These findings suggest that the Japanese version of the IPASE produces reliable and valid scores and is a clinically useful self‐report measure for assessing basic self‐disturbance.

This study had several limitations. First, test‐retest reliability was not assessed. Therefore, the temporal stability of the Japanese version of the IPASE remains to be established. Future studies should evaluate test‐retest reliability in clinically stable patients with schizophrenia and healthy controls. Second, the diagnosis of schizophrenia and the exclusion of psychiatric disorders in healthy controls were based on clinical judgment rather than structured interviews like the Structured Clinical Interview for DSM Disorders (SCID) or the Mini‐International Neuropsychiatric Interview (MINI). Although all participating psychiatrists were experts with extensive experience in diagnosing and treating schizophrenia, subjective judgment may have influenced the diagnostic process. Third, the schizophrenia group included participants with comorbid conditions, so the potential influence of these comorbidities on the results cannot be ruled out. Fourth, as standardized intelligence tests were not administered to either group, differences in IQ may have affected participants' understanding of the questionnaire items and, consequently, the results. Fifth, as the study was conducted at only two university hospitals, the sample may not be representative of the general clinical population. Future multicenter studies with larger sample sizes are needed to further examine the generalizability of this scale.

Despite these limitations, our findings indicate that the Japanese version of the IPASE is a highly reliable and valid instrument with practical applicability in both research and clinical settings. In particular, it contributes to advancing research on basic self‐disturbance from the perspective of phenomenological psychopathology and is expected to facilitate further progress in elucidating the pathophysiology of schizophrenia. Furthermore, it may serve as a useful screening tool in clinical settings where conducting detailed and time‐consuming interviews is challenging, thereby contributing to the diagnosis and treatment of schizophrenia.

## Author Contributions

**Dan Nakamura:** conceptualization, methodology, data curation, investigation, validation, formal analysis, writing – original draft, visualization, project administration. **Masafumi Mizuno:** conceptualization, methodology, supervision, investigation, project administration, writing – review and editing. **David Cicero:** validation, supervision, writing – review and editing. **Yu Arai:** investigation, project administration, writing – review and editing. **Hirohisa Suzuki:** investigation, writing – review and editing. **Shunsuke Hirose:** investigation, writing – review and editing. **Takahisa Kasai:** investigation, writing – review and editing. **Shutaro Sugita:** investigation, writing – review and editing. **Yoshifumi Nakamura:** formal analysis, writing – review and editing. **Stephen Wood:** supervision, writing – review and editing. **Andrea Polari:** supervision, writing – review and editing. **Takahiro Nemoto**: project administration, writing – review and editing. **Barnaby Nelson:** conceptualization, supervision, methodology, writing – review & editing.

## Funding

This study and the publication fee were supported by departmental research funds at Showa Medical University.

## Ethics Statement

This study was approved by the Showa Medical University Research Ethics Review Board (Approval No. 2024‐252‐A), and the protocols were conducted in accordance with the Declaration of Helsinki.

## Consent

All participants received a detailed explanation of the study procedures and provided written informed consent. For participants who were minors, the study was explained to both the participant and their legal guardian, and written informed consent was obtained from the guardian in addition to the participant's assent.

## Conflicts of Interest

The authors declare no conflicts of interest.

## Data Availability

The data that support the findings of this study are not available due to privacy or ethical restrictions.
